# Disparate Presentations of Localized Cystic Disease of Kidney: A Review with an Objective of Correct Approach for Accurate Treatment Plan

**DOI:** 10.7759/cureus.1187

**Published:** 2017-04-22

**Authors:** Sachin Khanduri, Mriganki Chaudhary, Tushar Sabharwal, Aakshit Goyal, Gaurav Katyal

**Affiliations:** 1 Radiodiagnosis, Era's Lucknow Medical College and Hospital

**Keywords:** autosomal dominant polycystic kidney disease, multicystic nephroma, cystic renal cell carcinoma, localised cystic disease of kidney

## Abstract

**Background:**

Localized cystic disease of the kidney is a rare, non-familial condition. Its imaging and clinical features are unique and need to be differentiated from autosomal dominant polycystic kidney disease and focal cystic masses such as multicystic nephroma and cystic renal cell carcinoma. It is always restricted to one kidney and is characterized by multiple cysts of varying sizes separated by residual normal renal tissue.

**Materials and methods:**

This study reports 12 cases of localized cystic disease of the kidney based on imaging findings and clinical histories. The modalities of choice were ultrasonography followed by contrast-enhanced computed tomography. Eight out of 12 patients were men and the average age of presentation was 46 years. The screening of family members and relatives was done to rule out the differentials.

**Results:**

Localized cystic disease of kidney was diagnosed in all the patients and it presented in two different forms. In three patients, multiple cysts involved whole of the kidney, resulting in thinned-out residual renal parenchyma. In the rest nine patients it remained localised to a particular segment of the kidney. No cysts were observed in the contralateral kidney in seven patients, and one or two simple cysts were observed in five. Clinical presentations included only flank pain in six patients, flank pain with palpable abdominal mass in four patients, two patients presented as asymptomatic cases with diagnosis as an incidental finding and one patient with hematuria. Eight patients underwent imaging and two underwent clinical follow-up for a period of two years showing stability of the disease. One patient underwent nephrectomy for suspected renal neoplasm.

**Conclusion:**

Localized cystic disease of the kidney is a unilateral, rare and stable disease that has two different forms of presentations. Its imaging findings should be clearly understood so as to not classify it as a separate disease and avoid unnecessary surgery. It rarely leads to hypertension or polycythemia, and until then no definitive management is required. It can be followed up using imaging techniques and requires nephrectomy only when the suspicion of malignancy is strong.

## Introduction

Localized cystic disease of kidney is a rare, non-familial, non-progressive and benign condition, first described as unilateral polycystic kidney disease in 1964. It is a multicystic disease characterized by cysts of varying sizes located in a diffusely enlarged kidney without forming a separately encapsulated mass [1-5]. Levine, et al. coined the term “Unilateral Polycystic Kidney Disease” in 1989. All the studies done before 1970 only reported that unilateral renal cystic disease (URCD) might be different from autosomal dominant polycystic kidney disease (ADPKD) [6]. It has been known by a variety of different names, most common being unilateral polycystic kidney disease and segmental polycystic kidney disease. The gross and histological appearances of localised cystic disease of kidney are very close to that of autosomal dominant polycystic kidney disease and it was speculated that the pathogenesis of both is similar; however, recent studies have proven them to be completely separate entities. Unilateral renal localization, absence of family history, absent extra-renal manifestations and non-progression towards chronic renal failure are some of the features that distinguish unilateral renal cystic disease (URCD) from autosomal dominant polycystic kidney disease (ADPKD).

## Materials and methods

Clinical and imaging profiles of twelve patients (obtained between December 2014-January 2016) were available for the study. All of these patients were evaluated at Era's Lucknow Medical College & Hospital and a diagnosis of localized cystic disease of kidney was made on the basis of the characteristic pattern of the disease on imaging studies and as described in the literature.

All the patients were examined with ultrasonography and computed tomography (CT). Computed tomography scan was performed through the upper abdomen using thin sections (5 mm) before and after contrast administration. The clinical profile of patients included age, sex, presenting symptoms, positive or negative family history, intervention performed if any, and the period of follow-up. Informed consent from all the patients and approval from the Ethical committee of Era's Lucknow Medical College & Hospital were obtained.

## Results

The clinical and imaging findings are summarized in Table 1.

**Table 1:** Table representing clinical and imaging findings.

Patient No.	Sex	Age (Yrs.)	Clinical Features	Site	Normal Kidney Remaining (%)	Other Kidney	Intervention	Follow-up (Yrs.)
1	M	32	Asymptomatic	Lower Pole	60	-	None	2
2	M	48	Flank Pain	UP & LP	30	Scattered Cysts	None	2
3	M	38	Flank Pain	Upper Pole	70	-	None	2
4	F	39	Flank Pain & Abdominal Mass	Entire Kidney	10	-	None	2
5	M	48	Flank Pain	UP & LP	30	-	None	Lost To Follow-up
6	M	30	Asymptomatic	Lower Pole	60	-	None	2
7	F	50	Flank Pain & Abdominal Mass	Entire Kidney	5	Scattered Cysts	None	2
8	M	39	Flank Pain	Upper Pole	70	-	None	2
9	M	49	Flank Pain & Abdominal Mass	MP & LP	30	Scattered Cysts	None	2
10	F	41	Flank Pain	Entire Kidney	5-10	Scattered Cysts	None	2
11	M	52	Flank Pain	UP & MP	30	Scattered Cysts	Nephrectomy	-
12	F	33	Flank Pain	Lower Pole	60	-	None	2

### Clinical features

Eight patients were men and four women. The age group of the patients was between 26-52 years and the average age of presentation was 46 years. Clinical presentations included only flank pain in six patients, flank pain with palpable abdominal mass in four patients, and two patients presented as asymptomatic cases with diagnosis as an incidental finding. There was no history of gastrointestinal, cardiovascular, or urological symptoms in any of the patients.

All the preliminary investigations-blood pressure, specific gravity of urine, hemoglobin, RBC count, total WBC count, platelet count, serum urea, serum creatinine and blood urea nitrogen along with echocardiography and colon examination were normal. Family history did not reveal any other member as having similar complaints.

On the basis of imaging findings, four patients were initially suspected of having a renal neoplasm, three were suspected of having autosomal dominant polycystic kidney disease; however, the family history was not supportive and five were diagnosed as having localized cystic kidney disease.

Ten patients underwent imaging follow-up for a period of two years. One of the patients suspicious for renal neoplasm underwent nephrectomy at an outside institution. In this patient, cysts involved the right kidney in the upper pole. Cysts were not encapsulated and pathology showed multiple cysts lined by flattened epithelium. They contained clear yellow fluid with no evidence of papillary formation and areas of normal or atrophic renal tissue separating them on cross-section. One patient was lost to follow-up.

### Ultrasound findings

**Figure 1:**
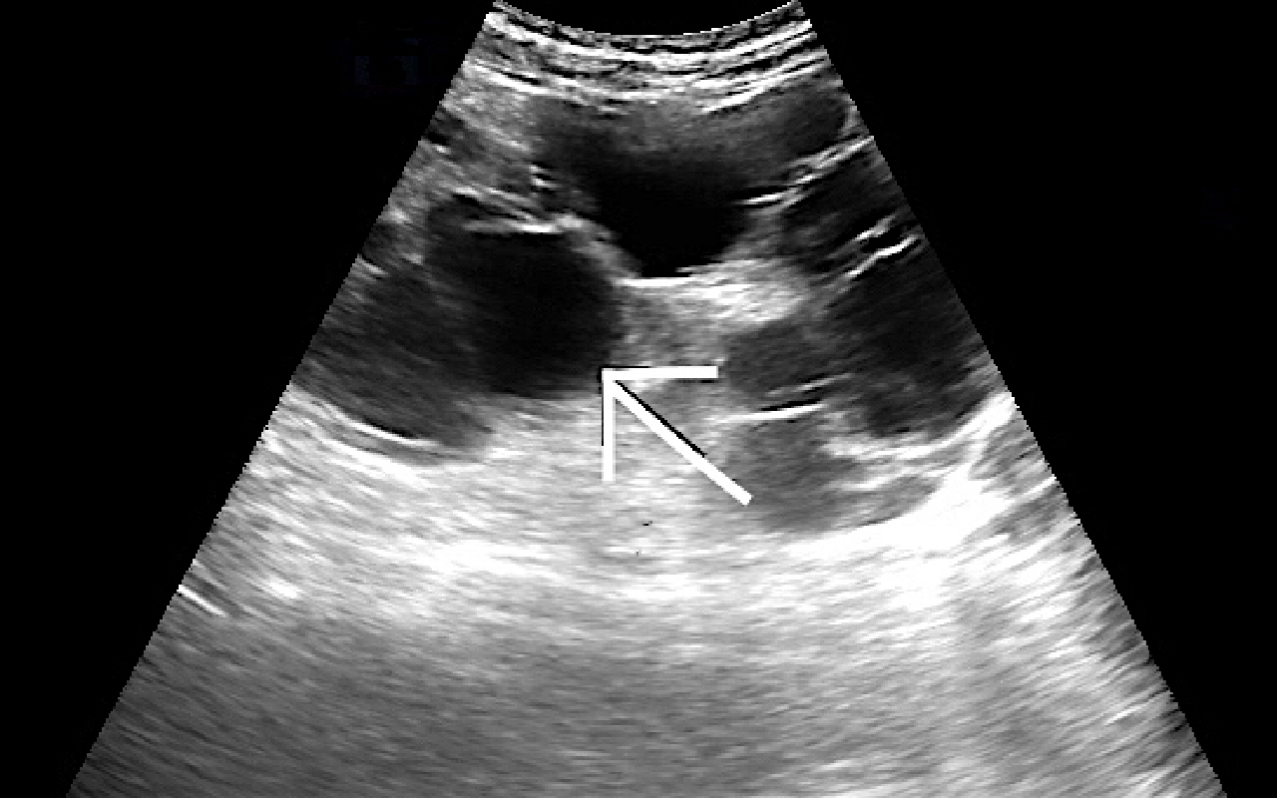
A 39-year-old male patient with complaints of left flank pain and abdominal distension. Ultrasound image of the affected left kidney shows multiple anechoic cysts (white arrow).

Renal ultrasonography in three cases revealed enlarged unilateral kidney with lobulated renal outline, with the entire kidney replaced by multiple non-specific anechoic cystic masses of varying sizes, separated by thin septae representing thinned-out residual renal parenchyma (Figure 1).

The remaining nine cases revealed similar presentation of the cysts; however, they were confined to a particular segment of the kidney, involving either of the poles.

### Computed tomography findings

**Figure 2:**
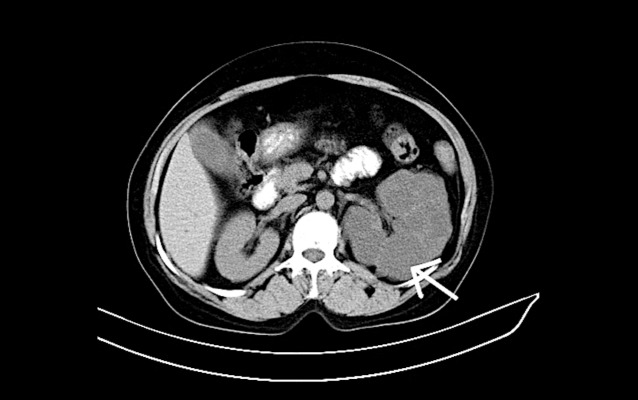
A 39-year-old male patient with complaints of left flank pain and abdominal distension. Unenhanced axial computed tomography (CT) scan of the left kidney shows multiple hypodense cysts separated by thinned out residual renal parenchyma (white arrow).

Non-contrast computed tomography findings revealed multiple hypodense simple cysts replacing almost the entire kidney with unenhanced thinned-out residual renal parenchyma (Figure 2).

**Figure 3:**
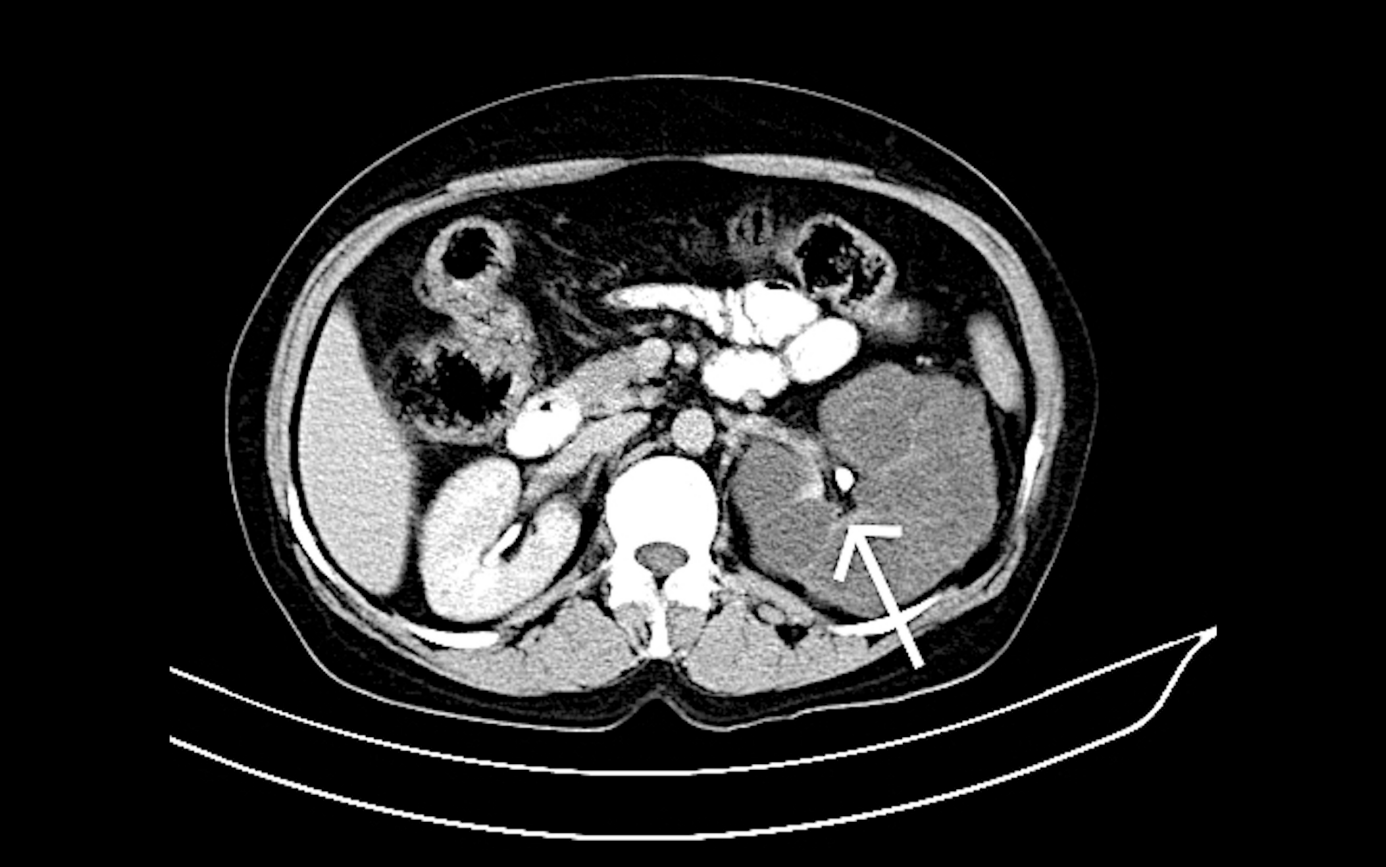
A 39-year-old male patient with complaints of left flank pain and abdominal distension. Intravenous contrast-enhanced axial computed tomography scan section of the left kidney shows multiple simple unenhanced hypodense cysts separated by contrast-enhanced thinned out residual renal tissue (white arrow).

Contrast-enhanced computed tomography findings revealed non-enhancing hypodense cystic masses, the normal thinned out residual renal parenchyma showing normal enhancement pattern with no evidence of any abnormally enhancing lesion (Figure 3).

**Figure 4:**
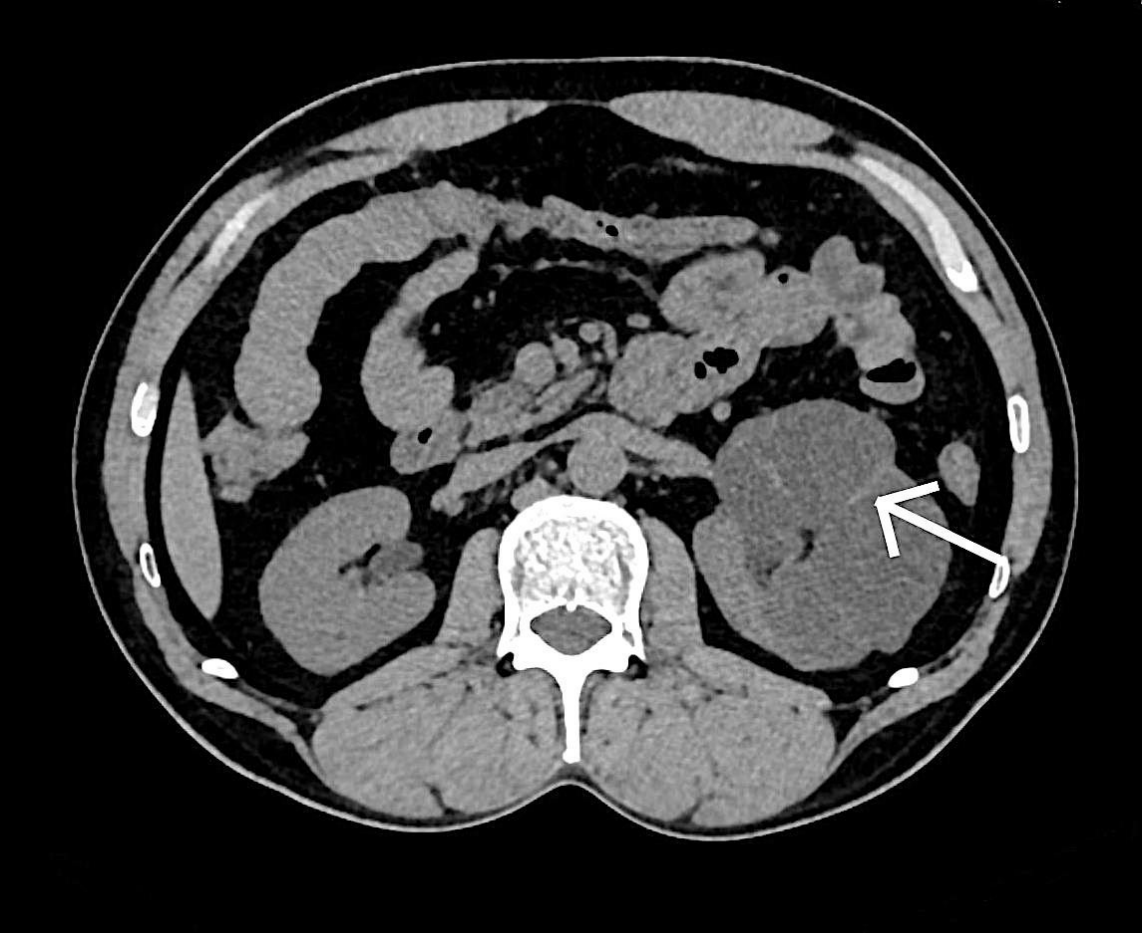
A 41-year-old female patient with complaint of left flank pain. Unenhanced axial computed tomography scan of the affected left kidney shows multiple hypodense cysts separated by thinned out residual renal parenchyma (white arrow).

**Figure 5:**
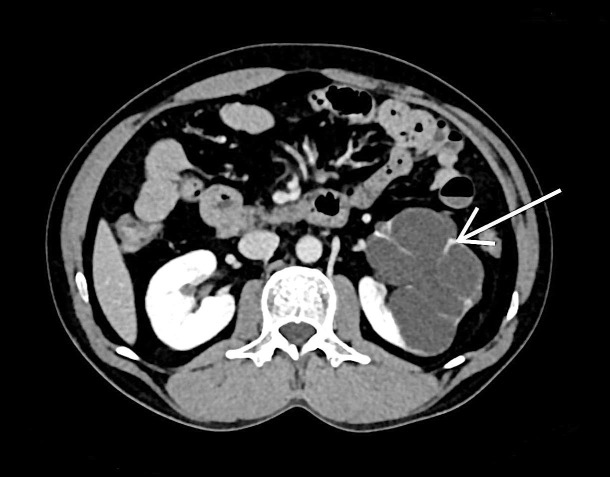
A 41-year-old female patient with complaint of left flank pain. Intravenous contrast-enhanced axial computed tomography scan section of the affected left kidney shows multiple simple unenhanced hypodense cysts separated by contrast-enhanced thinned out residual renal parenchyma (white arrow).

Para-renal fascia and renal capsule were completely intact with no evidence of either fat stranding or collection in the surrounding area. The contralateral kidney was seen to be normal in size and shape with maintained cortico-medullary differentiation and normally enhancing parenchyma in seven of the cases. It showed one/two small simple cysts in five cases. In the segmental involvement, non-contrast computed tomography findings revealed multiple hypodense simple cysts replacing the entire middle pole with unenhanced thinned out residual renal parenchyma (Figures 4-5).

**Figure 6:**
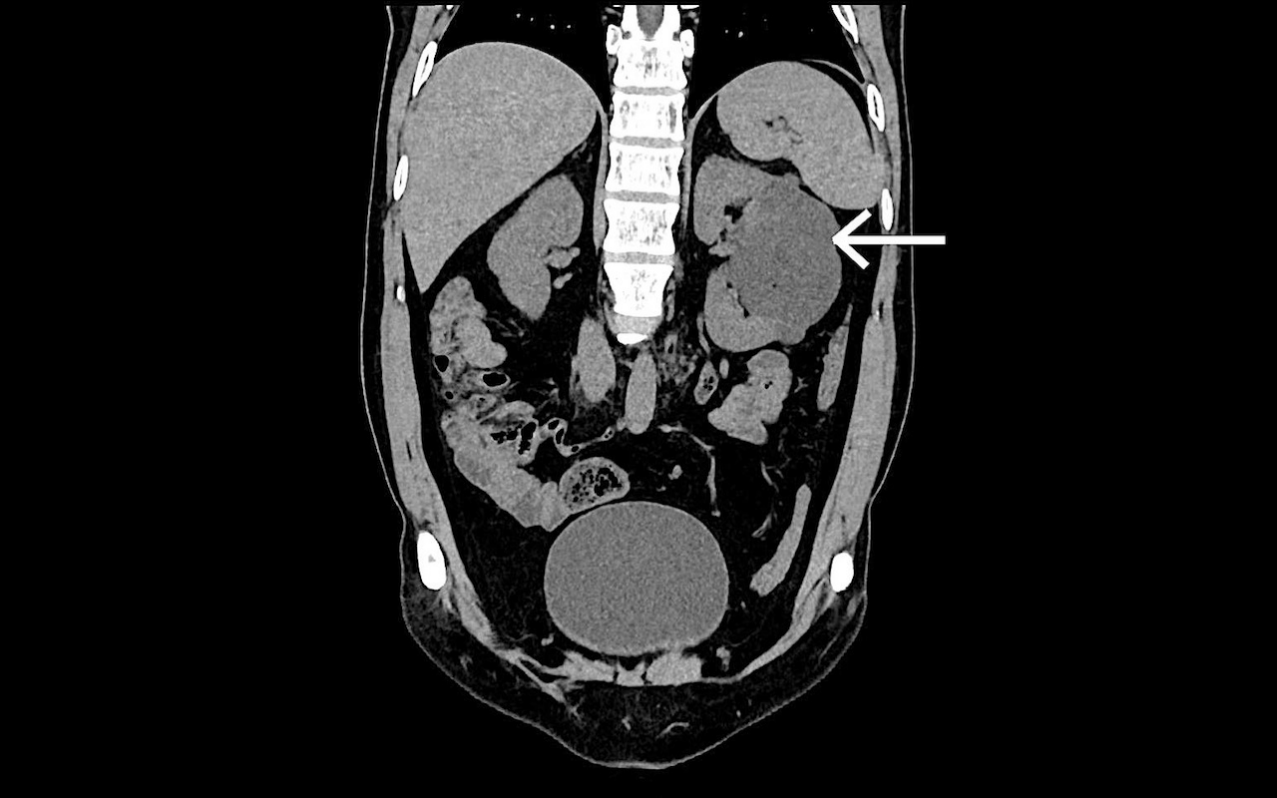
A 41-year-old female patient with complaint of left flank pain. Unenhanced coronal computed tomography scan of the affected left kidney shows multiple hypodense cysts separated by thinned out residual renal parenchyma (white arrow).

**Figure 7:**
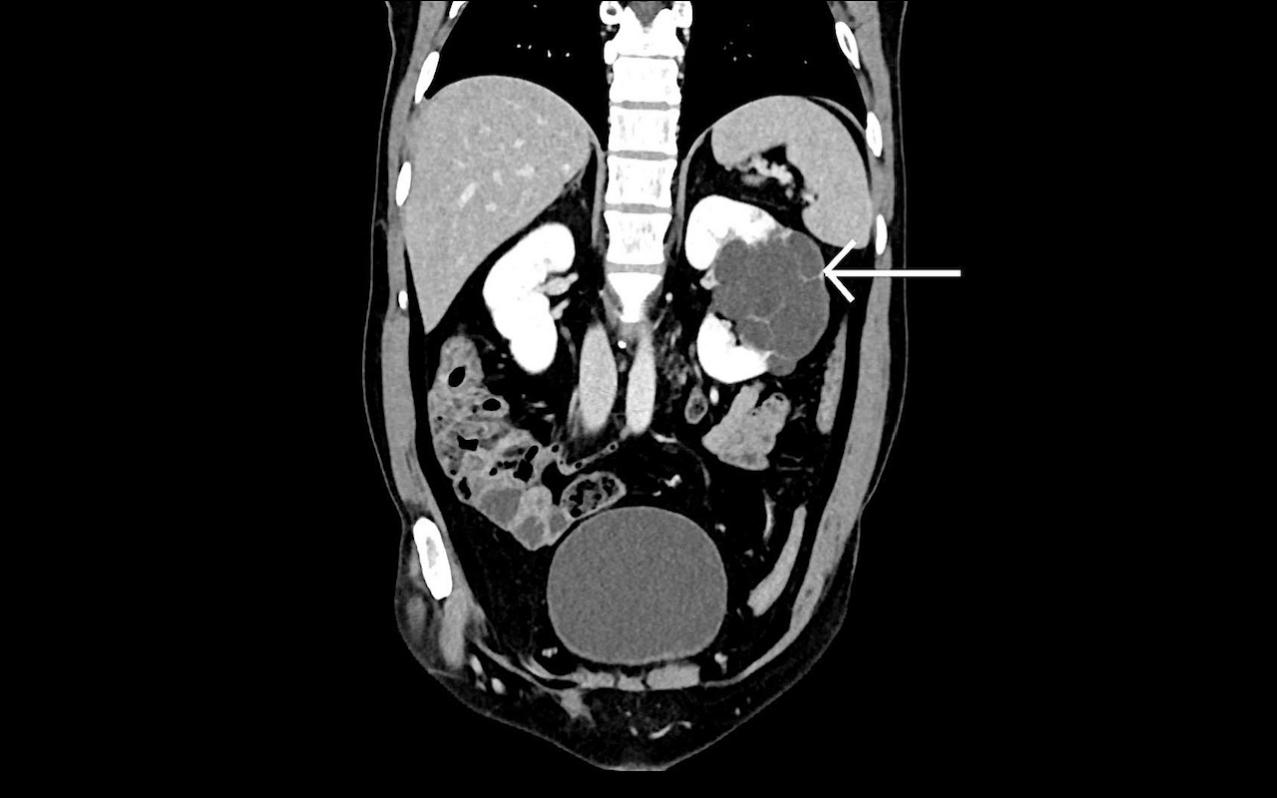
A 41-year-old female patient with complaint of left flank pain. Intravenous contrast-enhanced coronal computed tomography scan section of the affected left kidney shows multiple simple unenhanced hypodense cysts separated by contrast-enhanced thinned out residual renal parenchyma (white arrow).

Contrast-enhanced computed tomography findings revealed non-enhancing hypodense cystic masses, the normal thinned out residual renal parenchyma showing normal enhancement pattern with no evidence of any abnormally enhancing lesion (Figures 6-7).

Computed tomography findings of all the patients were characteristic of localized cystic disease of kidney due to their nature of either complete or segmental replacement of the kidney by a conglomerate mass separated by thinned-out normal/atrophic renal parenchyma.

The extent of involvement in all the patients varied considerably; however, unilateral involvement was noted in all the cases (Table 1). The entire kidney was involved in three patients with one/two cysts in the contralateral kidney, and a possibility of polycystic kidney disease was thought of, but there was no cystic involvement of any other organ and the family history was also negative. Nine patients had localized involvement of the disease with cysts limited to a particular segment of the kidney. Three patients showed involvement of the kidney’s lower pole. Three other patients showed involvement of the upper and middle poles. Two patients showed involvement of the upper pole with sparing of the mid and lower portions. One patient showed involvement of the middle and lower poles with sparing of the upper portion. The contralateral kidney was normal in seven patients with one/two small simple cysts in five patients older than 47 years.

Ten patients who had imaging/clinical follow-up maintained a good renal function for a period of two years proven by serial ultrasound examinations and renal function tests, documenting stability of the disease.

## Discussion

The pathogenesis of localized cystic disease is unknown but may represent an acquired condition [7]. Although, a select single axial image of localized cystic disease may potentially be confused with cystic neoplasm. Systematic evaluation of multiple sequential axial images should enable differentiation on the basis of the presence of a continuum of adjacent cysts rather than a focal encapsulated loculated mass [8]. Because of the difficulty of differentiating multilocular cystic nephroma from cystic Wilms tumor and multicystic renal cell carcinoma on the basis of imaging findings, patients are usually subjected to unnecessary surgery. Thus, imaging findings must be clearly understood as they lead to an appropriate clinical management.

Localized cystic disease of the kidney (LCDK) is a rare, non-familial, non-progressive renal pathology, not associated with extra-renal complications. Morphologically, it resembles ADPKD, which on the contrary shows progression towards renal failure and has extra-renal complications in the form of hepatic and pancreatic cysts, berry aneurysms, aortic dissections and bowel pathologies [9, 10]. In children, autosomal dominant polycystic kidney disease may manifest as localized cystic disease of kidney, but careful history along with radiological examinations of their parents leads to the diagnosis of autosomal dominant polycystic kidney disease. Computed tomography with its characteristic findings is one of the best modalities in diagnosing the unilateral localization of lesions and has avoided the need of surgical confirmation. Earlier studies have also reported confirmation of diagnosis by examining the nephrectomized specimens on autopsy [11].

Some of the other differential diagnoses such as cystic renal cell carcinoma, multicystic nephroma, multicystic dysplastic kidney and mutiple simple renal cysts are few other diseases that need to be differentiated from localized cystic disease of the kidney.

Cystic renal cell carcinoma grows slowly and usually forms discrete, encapsulated masses that are well demarcated and expand to displace the normal renal parenchyma, without containing the islands of enhancing renal parenchyma on computed tomography as seen in localized cystic disease of the kidney.

Multilocular cystic renal cell carcinoma cannot be clinically reliably distinguished from multicystic nephroma neither by physical examination nor by radiologic evaluation. Previous studies have indicated that even immunohistochemistry is unable to differentiate between these conditions [12-13]. However, unlike these entities, localized cystic disease of the kidney shows other cysts nearby that are clearly separate from the main conglomerate mass of cysts with no discrete encapsulation [14].

A multicystic dysplastic kidney is a renal pathology usually seen in infants and children but can also be seen in adults. The affected kidney shows poor excretory function due to ureteropelvic occlusion. On imaging, the kidney appears diffusely cystic and severely dysplastic. The dysplastic core of tissue may enhance after intravenous (IV) contrast medium administration but has a different nephrographic appearance from that of normal renal tissue. The collecting system draining the dysplastic segment appears atretic or obstructed, and therefore, is not usually opacified on contrast-enhanced computed tomography. On the other hand, in localized cystic kidney disease the collecting system shows only displacement with unaltered renal excretion and is thus easily distinguishable using computed tomography and radionuclide studies. The tissue between the cysts in localized cystic disease is normal (or atrophic) rather than dysplastic.

It may be difficult to differentiate multiple simple renal cysts from unilateral renal cystic disease (when confined to one kidney); however, the cysts are not as numerous as in URCD.

In our study, ultrasonography (USG) findings correlated with those of CT findings. Nine cases revealed anechoic cysts localized to one kidney and three were seen diffusely involving unilateral kidney which on CT were seen as non-enhancing, non-encapsulated hypodense lesions with normally enhancing intervening renal parenchyma. All the patients on two year follow up revealed clinical and radiological stability.

## Conclusions

Localized cystic disease of the kidney is an entity that needs to be recognized as a benign, stable, non-surgical condition. It requires periodic follow-up of the patients using imaging techniques such as ultrasonography and computed tomography. In-depth knowledge of this disease is necessary in order to clearly differentiate it from ADPKD, cystic nephroma, cystic renal cell carcinomas and multicystic dysplastic kidney as all of these close differentials have different clinical and surgical approach for treatment. This is important to avoid unnecessary nephrectomy.
